# Supplementary material for: Blocking muscle wasting via deletion of the muscle specific E3 ubiquitin ligase MuRF1 impedes pancreatic tumor growth
Source: Res Sq. 2023 Feb 9:rs.3.rs-2524562. Preprint. [Version 1] doi: 10.21203/rs.3.rs-2524562/v1 (PMC9934780; doi:10.21203/rs.3.rs-2524562/v1)
Supplement: 1 [file NIHPPRS2524562V1-supplement-1.pdf]

A

Best fitted values	Sham	WT KPC D14-15	p-value
Y:	16.38	9.98	<0.0001
K:	0.016	0.016	0.9660
Plateau:	5.24	4.32	0.0010

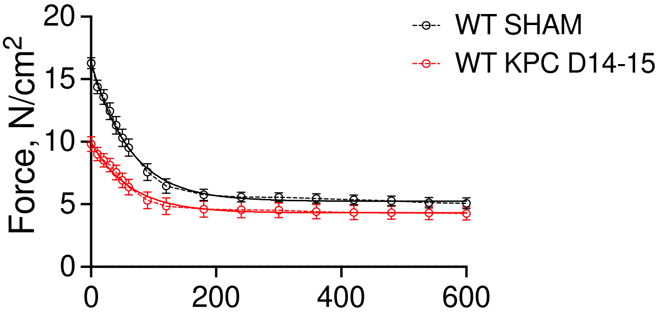

B

Best fitted values	MuRF1$^{-/-}$Sham	MuRF1$^{-/-}$ KPC D14-15	MuRF1$^{-/-}$ KPC D25-27	p-value
Y:	16.26	16.48	14.61	0.0051
K:	0.011	0.012	0.013	0.6051
Plateau:	5.13	5.20	4.39	0.0477

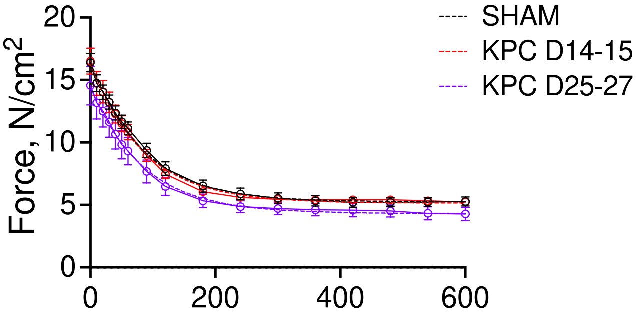

Supplementary Figure 1. MuRF1 deletion protects against tumor-induced reduction in *soleus* evoked forces during a fatiguing protocol. Evoked force data from WT mice (**A**) and MuRF1$^{-/-}$ mice **(B)** were fitted with an exponential one-phase decay model (i.e. Y=(Y0 - Plateau)*exp(-K*X) + Plateau). Y = initial evoked force, K = decay rate constant and Plateau = evoked forces at infinite time. Dashed lines depicts the one-phase decay model curves.

A

Ubiquitination site	WT D8	WT D10	WT D12	WT D14	WT END	MuRF1-/- D12	MuRF1-/- D16	MuRF1-/- END	MuRF1 OE
desmin_308	2.1	2.7	2.5	2.0	1.6	-1.8	-2.1	-2.9	2.7
GAPDH_143	2.7	-1.0	1.4	1.0	1.5	-3.0	1.8	1.7	11.2
LDH-A_243	-1.7	-6.0	6.8	3.4	2.6	-5.8	-6.3	-4.4	3.5
MYH4_924	-1.7	-6.0	6.8	3.4	2.6	ND	ND	ND	3.9
MYLPF_105	-2.8	-1.9	1.3	2.2	-1.3	-2.0	-1.5	-1.2	2.6
MYLPF_112	-1.2	1.4	2.7	3.6	3.2	-1.5	-1.2	-1.9	4.8
SQSTIM1_13	1.1	8.8	6.4	26.8	48.0	-3.8	-4.1	-2.9	3.2
TNNT3_75	1.0	3.9	9.9	24.5	26.4	-2.0	-1.4	-1.3	5.1
MURF1_123	2.9	1.0	63.7	95.0	246.6	ND	ND	ND	45.3
MURF1_199	1.1	1.0	8.3	9.0	10.5	ND	ND	ND	123.6
MURF1_255	-1.3	-1.2	8.2	7.3	8.0	ND	ND	ND	212.3
titin_21631	1.1	1.2	-1.1	2.6	3.3	-13.5	-2.0	-2.9	5.7
titin_21921	1.2	-1.1	2.0	2.6	2.5	-1.3	-1.2	-1.1	3.2
titin_21944	1.8	1.6	1.8	5.6	5.2	-1.2	1.0	-1.2	61.4
titin_27043	-1.1	-1.1	1.8	4.4	4.9	-3.6	-3.9	-2.8	3.1
titin_27236	1.6	2.6	1.8	3.7	2.8	-2.4	-2.1	-5.6	3.5
titin_27460	-2.2	-1.3	1.1	2.6	3.6	-1.3	1.3	-1.9	9.2
titin_27654	-2.3	-2.0	4.9	21.5	14.9	-1.3	1.0	-4.6	3.6
titin_30935	2.3	2.7	5.0	10.4	9.9	-1.5	-1.1	-1.4	3.9
titin_32635	1.1	2.1	9.2	19.7	18.2	-6.7	-5.1	-7.3	8.1
VCP_231	-1.0	1.2	3.1	6.0	5.0	-1.3	-1.3	-1.5	4.7

Supplementary Figure 2. Sites showing increased ubiquitination in response to KPC tumor burden and MuRF1 overexpression (Baerh et al. 2021). Red indicates increased ubiquitination levels and blue indicates reduced ubiquitination levels. ND = not detected.

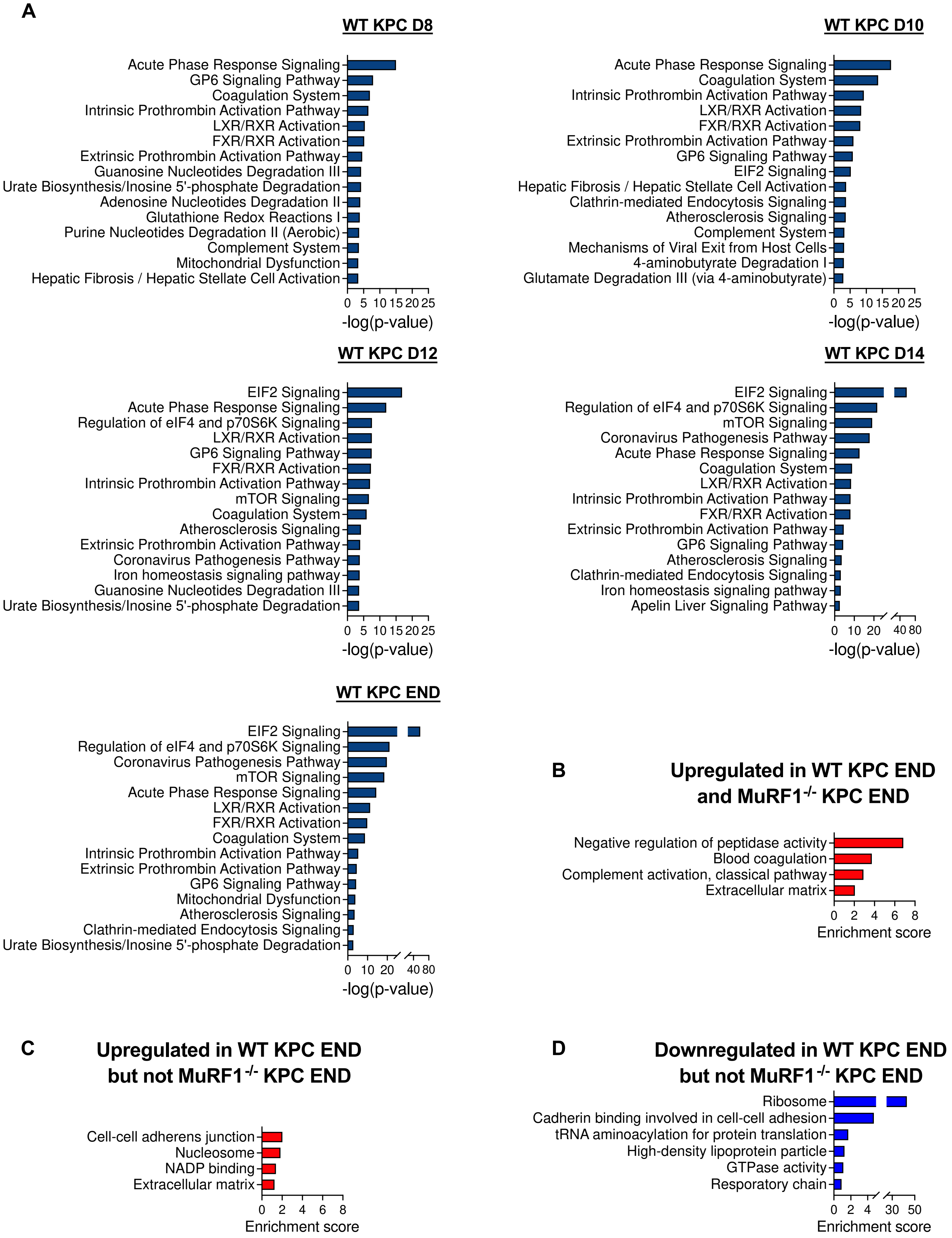
A
WT KPC D8
Acute Phase Response Signaling
GP6 Signaling Pathway
Coagulation System
Intrinsic Prothrombin Activation Pathway
LXR/RXR Activation
FXR/RXR Activation
Extrinsic Prothrombin Activation Pathway
Guanosine Nucleotides Degradation III
Urate Biosynthesis/Inosine 5'-phosphate Degradation
Adenosine Nucleotides Degradation II
Glutathione Redox Reactions I
Purine Nucleotides Degradation II (Aerobic)
Complement System
Mitochondrial Dysfunction
Hepatic Fibrosis / Hepatic Stellate Cell Activation
0 5 10 15 20 25
-log(p-value)
WT KPC D10
Acute Phase Response Signaling
Coagulation System
Intrinsic Prothrombin Activation Pathway
LXR/RXR Activation
FXR/RXR Activation
Extrinsic Prothrombin Activation Pathway
GP6 Signaling Pathway
EIF2 Signaling
Hepatic Fibrosis / Hepatic Stellate Cell Activation
Clathrin-mediated Endocytosis Signaling
Atherosclerosis Signaling
Complement System
Mechanisms of Viral Exit from Host Cells
4-aminobutyrate Degradation I
Glutamate Degradation III (via 4-aminobutyrate)
0 5 10 15 20 25
-log(p-value)
WT KPC D12
EIF2 Signaling
Acute Phase Response Signaling
Regulation of eIF4 and p70S6K Signaling
LXR/RXR Activation
GP6 Signaling Pathway
FXR/RXR Activation
Intrinsic Prothrombin Activation Pathway
mTOR Signaling
Coagulation System
Atherosclerosis Signaling
Extrinsic Prothrombin Activation Pathway
Coronavirus Pathogenesis Pathway
Iron homeostasis signaling pathway
Guanosine Nucleotides Degradation III
Urate Biosynthesis/Inosine 5'-phosphate Degradation
0 5 10 15 20 25
-log(p-value)
WT KPC D14
EIF2 Signaling
Regulation of eIF4 and p70S6K Signaling
mTOR Signaling
Coronavirus Pathogenesis Pathway
Acute Phase Response Signaling
Coagulation System
LXR/RXR Activation
Intrinsic Prothrombin Activation Pathway
FXR/RXR Activation
Extrinsic Prothrombin Activation Pathway
GP6 Signaling Pathway
Atherosclerosis Signaling
Clathrin-mediated Endocytosis Signaling
Iron homeostasis signaling pathway
Apelin Liver Signaling Pathway
0 10 20 40 80
-log(p-value)
WT KPC END
EIF2 Signaling
Regulation of eIF4 and p70S6K Signaling
Coronavirus Pathogenesis Pathway
mTOR Signaling
Acute Phase Response Signaling
LXR/RXR Activation
FXR/RXR Activation
Coagulation System
Intrinsic Prothrombin Activation Pathway
Extrinsic Prothrombin Activation Pathway
GP6 Signaling Pathway
Mitochondrial Dysfunction
Atherosclerosis Signaling
Clathrin-mediated Endocytosis Signaling
Urate Biosynthesis/Inosine 5'-phosphate Degradation
0 10 20 40 80
-log(p-value)
B
Upregulated in WT KPC END and MuRF1-/- KPC END
Negative regulation of peptidase activity
Blood coagulation
Complement activation, classical pathway
Extracellular matrix
0 2 4 6 8
Enrichment score
C
Upregulated in WT KPC END but not MuRF1-/- KPC END
Cell-cell adherens junction
Nucleosome
NADP binding
Extracellular matrix
0 2 4 6 8
Enrichment score
D
Downregulated in WT KPC END but not MuRF1-/- KPC END
Ribosome
Cadherin binding involved in cell-cell adhesion
tRNA aminoacylation for protein translation
High-density lipoprotein particle
GTPase activity
Resporatory chain
0 2 4 30 50
Enrichment score

Supplementary Figure 3. Bioinformatic enrichment analyses of skeletal muscle proteins showing altered abundance in response to tumor burden in the presence and absence of MURF1. (**A**) Canonical pathway enrichment analyses conducted on the skeletal muscle proteome of wild-type (WT) mice on day 8, 10, 12, 14 and 16 (END) post-KPC cell inoculation. (**B**) GO and KEGG terms enrichment analyses of proteins showing *increased* abundance in WT KPC END **and** MuRF1$^{-/-}$ KPC END. (**C**) GO and KEGG terms enrichment analyses of proteins showing *increased* abundance in WT KPC END **but not in** MuRF1$^{-/-}$ KPC END. (**D**) GO and KEGG terms enrichment analyses of proteins showing *decreased* abundance in WT KPC END **but not in** MuRF1$^{-/-}$ KPC END.
